# Angiographic features of drug-induced bilateral angle closure and transient myopia with Ciliochoroidal effusion

**DOI:** 10.1186/s12886-019-1230-y

**Published:** 2019-11-04

**Authors:** Yong Koo Kang, Byeong Jae Son, Dong Ho Park, Jae Pil Shin

**Affiliations:** Department of Ophthalmology, School of Medicine, Kyungpook National University, 130 Dongdeok-ro, Jung-gu, Daegu, 41944 South Korea

**Keywords:** Ciliochoroidal effusion, Drug-induced angle closure, Indocyanine green angiography, Transient myopia

## Abstract

**Background:**

To report five cases of acute drug-induced angle closure and transient myopia with ciliochoroidal effusion and to analyze angiographic findings of these cases.

**Methods:**

This study is an observational case series. Five patients with acute drug-induced angle closure and transient myopia with ciliochoroidal effusion were examined by fluorescein angiography, indocyanine green angiography (ICGA) and ultrasound biomicroscopy (UBM).

**Results:**

Five patients presented with bilateral visual loss and ocular pain after intake of topiramate, methazolamide, phendimetrazine tartrate or mefenamic acid. All patients showed elevated intraocular pressure (IOP) with shallow anterior chamber and myopic shift from − 0.5 to − 17.0 diopters (D). UBM showed ciliochoroidal effusions with diffuse thickening of the ciliary body in all cases. Rapid normalization of IOP and decrease of myopic shift occurred in all patients after discontinuing the suspected drugs. We classified the ICGA findings into 2 major signs (hypofluorescent dark spots, hyperfluorescent pinpoints) and 3 minor signs (diffuse choroidal hyperfluorescence, early hyperfluorescence of choroidal stromal vessel, and leakage and dilated retinal vessels).

**Conclusions:**

The pathogenesis of acute drug-induced angle closure and transient myopia with ciliochoroidal effusion may be idiosyncratic reaction of uveal tissue to systemic drugs. Accumulation of extravascular fluid in the ciliochoroidal layer had a major role in the pathogenesis. ICGA could be a useful method to examine the pathophysiology of this condition by imaging of the choroidal layer.

## Background

Bilateral angle closure glaucoma with ciliochoroidal effusion and transient myopia is a well-known complication of sulfa drugs such as topiramate [1–3], hydrochlorothiazide [4–6], acetazolamide [6, 7], methazolamide [8, 9], and indapamide [10]. Recently, cases of bilateral angle closure glaucoma and transient myopia with ciliochoroidal effusion induced by mefenamic acid [11] or anorexiants, including phendimetrazine tartrate and ephedrine [12], have been reported. For drug-induced angle closure glaucoma with ciliochoroidal effusion, there is no known report of angiographic findings, including indocyanine green angiography (ICGA), although the main pathology of this condition is thought to be associated with alterations of the ciliochoroidal layer. ICGA should therefore be an effective method to evaluate choroidal changes in such cases.

In this report, we describe a series of five patients who presented with acute bilateral angle closure and transient myopia with ciliochoroidal effusion after use of topiramate, methazolamide, mefenamic acid and phendimetrazine tartrate. In addition, we summarize the characteristic features of angiographic findings of these patients and suggest the underlying pathogenesis of this condition.

## Methods

This is a retrospective observational case series from January 2011 to August 2016. This study adhered to the tenets of the Declaration of Helsinki, and the protocol was approved by the Institutional Review Board/Ethics Committee of Kyungpook National University Hospital (IRB No. 2018–08-014). Written informed consent was obtained from the patients or the parents of patients under the age of 18 for publication of this article and any accompanying images. A copy of the written consent is available for review by the Editor of this journal. Each patient was observed from the onset of symptoms to the normalization of clinical signs. Patients were examined using slit-lamp biomicroscopy, intraocular pressure (IOP) measurements, indirect ophthalmoscopy, ultrasound biomicroscopy (UBM), B-scan ultrasonography, fluorescein angiography (FA), and ICGA during the acute and convalescent stages. UBM and B-scan ultrasonography examination were performed using a HiScan® (Optikon, Rome, Italy). FA and ICGA were performed using a HRA2® (Heidelberg Engineering, Heidelberg, Germany). Systemic medications in each patient were reviewed at presentation.

We reviewed and analyzed angiographic findings during the acute stages of the disease, and subdivided into two classes: two major signs, which were always found in all patients, and three minor signs, which were less consistent. Angiographic findings were retrospectively analyzed using a standard protocol [13].

## Results

Five patients had bilateral decreased vision and ocular pain after taking suspected systemic medications. In all patients, slit-lamp examination revealed narrow central anterior chamber depth and peripheral anterior chamber depth less than one-fourth of the corneal thickness. The ranges of IOP were from 33 to 60 mmHg. Spherical equivalents showed myopic shifts from − 0.5 to − 17.0 diopters (D). UBM revealed ciliochoroidal effusion with diffuse thickening of the ciliary body in all patients. Rapid normalization of IOP occurred in all patients after discontinuing the suspected drugs and use of cycloplegics. We summarize characteristics of all cases in Table 1 and angiographic findings of each case is described below.

**Table 1:** Characteristics of each case in acute drug-induced bilateral angle closure and transient myopia with ciliochoroidal effusion

Case No.	Age (years)	Medication			BCVA (Snellen chart)		IOP (mmHg)		Spherical equivalents (Diopter)	
		Ingredients	Reason for prescription		Initial	Final	Initial	Final	Initial	Final
1	9	Mefenamic acid Formoterol	Common cold	OD	20/200	20/20	37	16	−15.0	−0.75
				OS	20/40	20/20	43	16	−15.0	−0.75
2	27	Phendimetrazine Ephedrine Herbal laxatives	Weight gain	OD	20/200	20/20	64	10	−17.0	−0.5
				OS	20/200	20/20	54	10	−14.0	−0.5
3	28	Topiramate Azosemide Ephedrine	Weight gain	OD	20/40	20/20	40	13	−8.0	−0.5
				OS	20/40	20/20	39	13	−8.0	−0.5
4	51	Methazolamide	Diabetic macular edema	OD	20/100	20/23	26	16	−0.5	0
				OS	20/40	20/25	41	16	−6.0	−2.0
5	35	Topiramate	Weight gain	OD	20/100	20/20	33	10	−17.0	−0.25
				OS	20/100	20/20	33	10	−14.0	−0.25

### Case 1

A 9-year-old female presented with bilateral decreased vision for 3 days. She had a history of taking medications including mefenamic acid and formoterol, which were prescribed for common cold. She had no allergy history. On presentation, her best-corrected visual acuity was 20/200 in the right eye and 20/40 in the left eye. IOP was 37 mmHg in her right eye and 43 mmHg in her left eye. She developed acute myopic shifts of − 15.0 D in both eyes. Systemic medications were discontinued, and she was treated with topical cycloplegics and topical antiglaucoma medications. On the next day, the IOP was normalized to 16 mmHg in both eyes. On day 4, spherical equivalents decreased to − 0.75 D in both eyes. Visual acuity was improved to 20/20 in both eyes after 1 month.

### Case 2

A 27-year-old female presented with bilateral ocular pain and blurry vision for 2 days. The symptoms began abruptly several days after taking anorexiant medications which included phendimetrazine tartrate, ephedrine, and herbal laxatives composed of aloe and cynara extracts. She had no history of medical allergies. At initial examination, her visual acuity was 20/200 in both eyes and IOP was 64 mmHg in the right eye and 54 mmHg in the left eye. She developed acute myopic shifts of − 17.0 D in her right eye and − 14.0 D in her left eye. The patient was instructed to discontinue all anorexiant medications, and topical cycloplegics and antiglaucoma medications were given. Three days after treatment, IOP was normalized to 10 mmHg in both eyes and spherical equivalents decreased to − 0.5 D in both eyes. One month later, visual acuity was 20/20 in both eyes without any other ocular symptoms.

### Case 3

A 28-year-old female presented with acute blurry vision in both eyes for 3 days. Her medications included oral topiramate, azosemide and ephedrine, which she started 1 week prior to her initial examination. Her medications were prescribed for weight loss by a local general practitioner. She had no history of medical allergies. At presentation, her visual acuity was 20/40 with − 8.0 D of myopic shift in both eyes. The IOP was 40 mmHg in her right eye and 39 mmHg in her left eye. Systemic medications were discontinued, and the patient was treated with topical cycloplegics and antiglaucoma medications. The next day, the IOP was normalized to 13 mmHg in both eyes. On day 5, spherical equivalents decreased to − 0.5 D in both eyes and visual acuity was improved to 20/20 in both eyes.

### Case 4

A 51-year-old male visited our clinic with blurry vision in both eyes. He used 100 mg per day of methazolamide for refractory diabetic macular edema. His right eye was pseudophakic and he had no medical allergies. Ten days after taking oral methazolamide, he experienced a sudden decrease of vision in both eyes. On initial examination, his visual acuity was 20/100 in his right eye and 20/40 in his left eye. The IOP was 26 mmHg in his right eye and 41 mmHg in his left eye. He developed acute myopic shifts of − 0.5 D in the right eye and − 6.0 D in the left eye. Methazolamide was discontinued, and the patient was treated with oral prednisolone, topical cycloplegics, and topical antiglaucoma medications. On the next day, the IOP was normalized to 16 mmHg in both eyes. On day 8, the spherical equivalents decreased to plano in the right eye, and − 2.0D in the left eye. Visual acuity was improved to 20/23 in his right eye and 20/25 in his left eye. This case has already been reported as a separate case report [8].

### Case 5

A 35-year-old female presented with bilateral decreased vision for 2 days. The symptoms began abruptly one week after taking anorexiant medications including topiramate prescribed by a local general practitioner. She had no history of medical allergies. On initial examination, best corrected visual acuity was 20/100 with − 6.0 D of myopic shift in both eyes and IOP was 33 mmHg in both eyes. All systemic medications were discontinued, and she was treated with topical cycloplegics and topical antiglaucoma medications. The next day, the IOP was normalized to 10 mmHg in both eyes. On day 4, spherical equivalents decreased to − 0.25 D in both eyes and visual acuity was improved to 20/20 in both eyes.

#### Angiographic findings

### Major signs

#### Hypofluorescent dark spots (HDS)

**Fig. 1:**
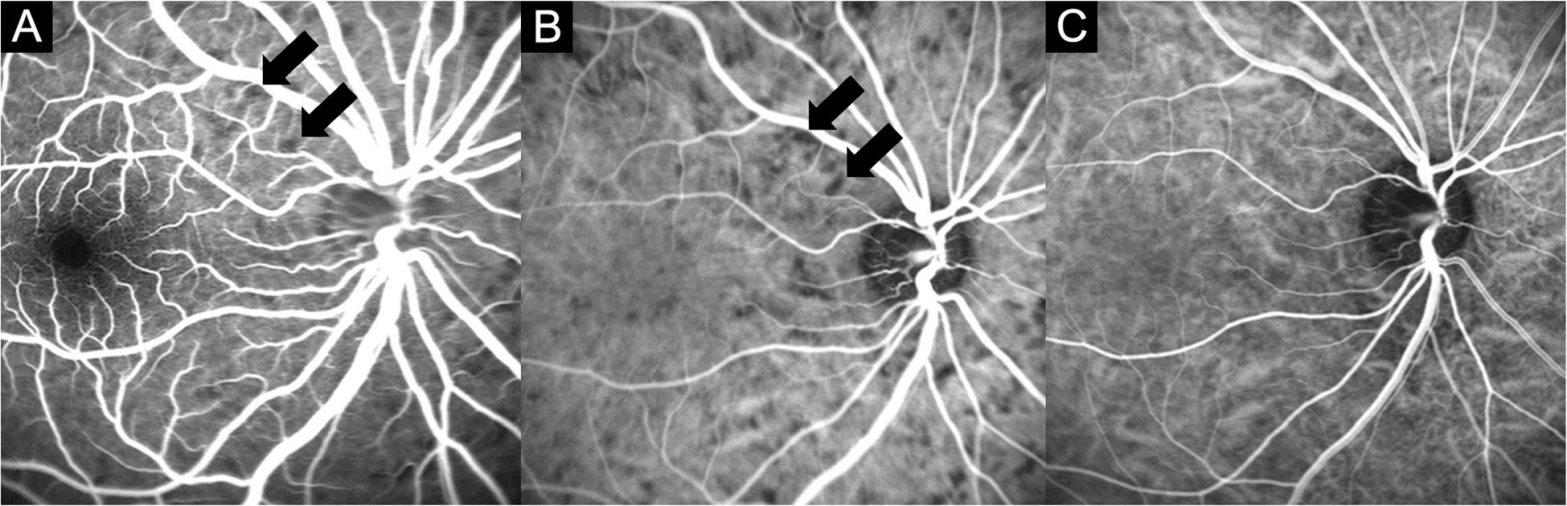
Fluorescein angiography (FA) and indocyanine green angiography (ICGA) of case 1. Hypofluorescent dark spots in the acute stage overlapped in the FA (**a**) and ICGA (**b**) (black arrows). **c** Hypofluorescent dark spots in the ICGA disappeared 1 month later

**Fig. 2:**
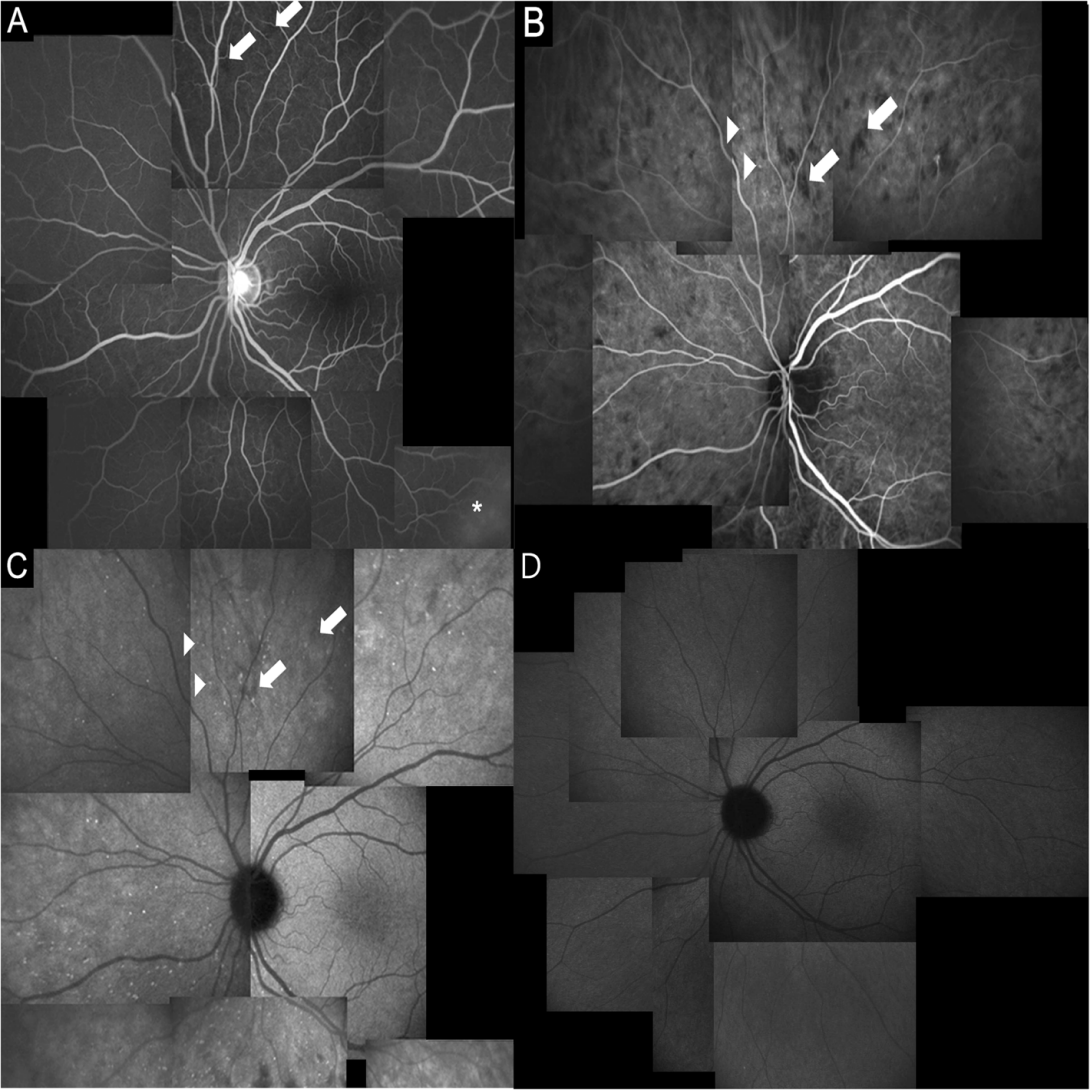
Fluorescein angiography (FA) and indocyanine green angiography (ICGA) of case 3. **a** FA shows hypofluorescent dark spots in the mid-peripheral retina (arrows) and retinal vascular leakage (asterisk) in the peripheral retina in the early phase. **b** In the early phase, ICGA shows that hypofluorescent dark spots are more prominent. Note the same location of hypofluorescent dark spots using FA and ICGA (arrows). Using ICGA, pinpoint hyperfluorescence appears near the hypofluorescent dark spots (arrowheads) in the early phase. **c** In the late phase, ICGA shows that some of the hyperfluorescent dark spots disappear, and pinpoint hyperfluorescence is more numerous. (D) The pinpoint hyperfluorescence in ICGA disappears after 1 month

FA showed HDS throughout the whole retina in the early phase (Figs. 1a, 2a). These spots were more prominent in ICGA (Figs. 1b, 2b, c). In ICGA, HDS appeared in the early phase and were the most prominent in the mid-peripheral retina. The HDS were small and round in the posterior pole but large and linearly shaped in the peripheral retina (Figs. 1b, 2b, c). During the late phase, some of the HDS in ICGA disappeared, but large HDS remained (Fig. 2c). Each spot overlapped in both FA and ICGA images but was not visible in the fundus images (Fig. 1a, b). The HDS were found in all cases and were completely normalized after 1 month (Fig. 1c).

#### Pinpoint hyperfluorescence

In ICGA, scattered hyperfluorescent pinpoints appearing in the early and midphase were seen in the mid-peripheral retinas (Fig. 2b). These hyperfluorescent pinpoints were more prominent in the late phase of ICGA but were not visible in the FA and did not show leakage (Fig. 2c). The lesions were predominantly located near HDS. These pinpoints were presented in all cases and were completely normalized after 1 month (Fig. 2d).

### Minor signs

**Fig. 3:**
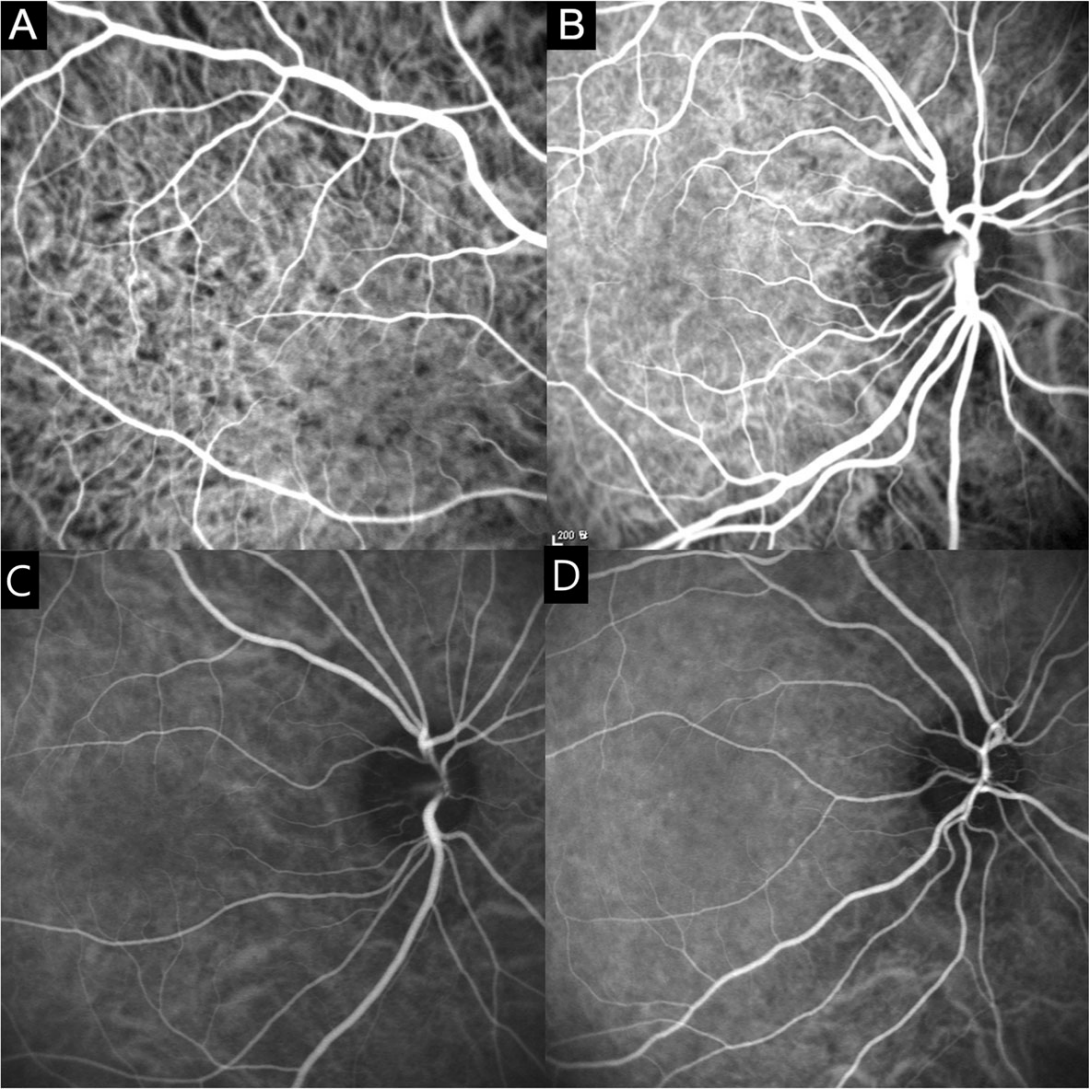
Indocyanine green angiography (ICGA) of the early phase of case 1 (**a**) and case 3 (**b**). There is early choroidal stromal vessel hyperfluorescence and leakage. Hyperfluorescence and leakage from choroidal stromal vessels disappear in the early phase ICGA of case 1 (**c**) and case 3 (**d**) after 1 month

***Early hyperfluorescence and leakage of choroidal stromal vessel:*** Hyperfluorescence and leakage from choroidal stromal vessels were seen in the early phase of ICGA of case 1 and 3 (Fig. 3a, b). These findings were disappeared after 1 month (Figure 3c, d).

**Fig. 4:**
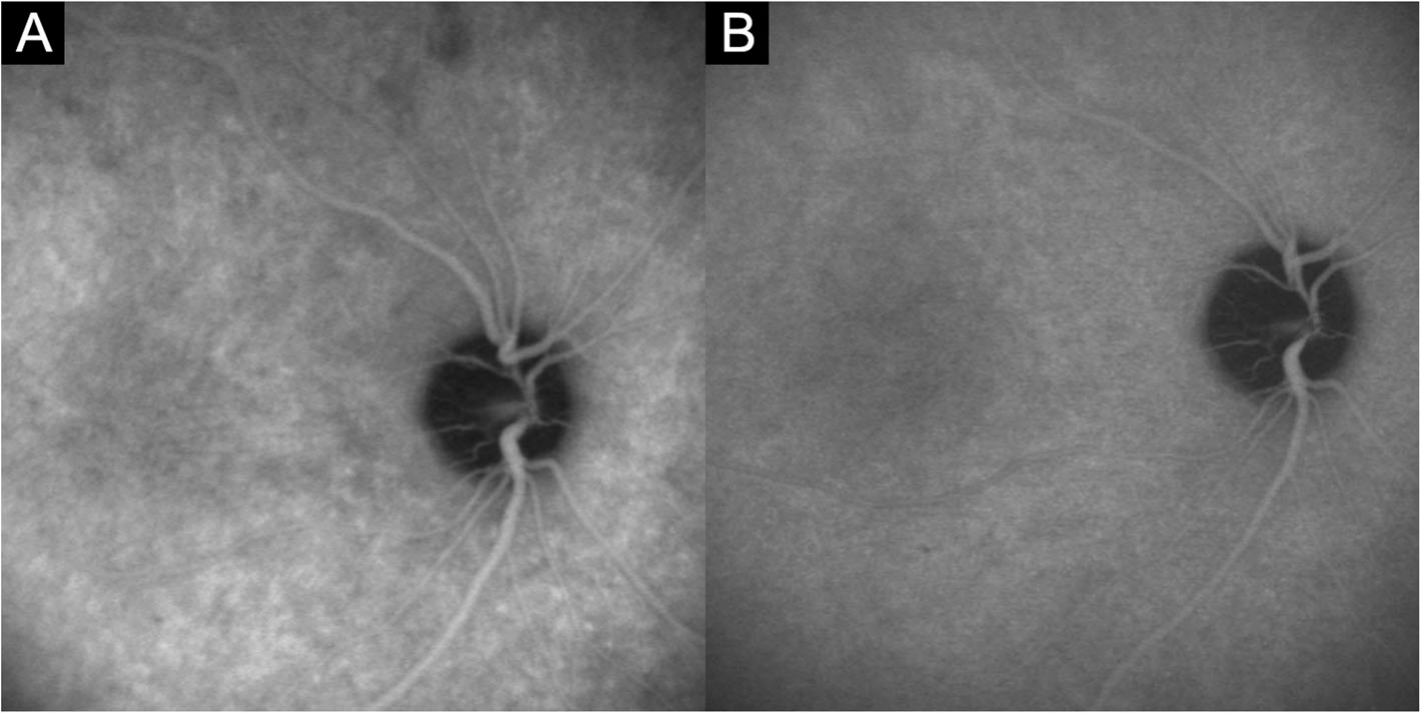
**a** Indocyanine green angiography (ICGA) of the intermediate phase (10 min and 24 s) in case 1, showing diffuse hyperfluorescence with pinpoint hyperfluorescence. **b** Indocyanine green angiography (ICGA) shows no diffuse hyperfluorescence in the intermediate phase (9 min and 42 s) after 1 month

***Diffuse choroidal hyperfluorescence in the intermediate phase*:** Diffuse hyperfluorescence of the choroid was seen in the intermediate phase of ICGA (about 10 min) in case 1 and 2 (Figure 4).

**Fig. 5:**
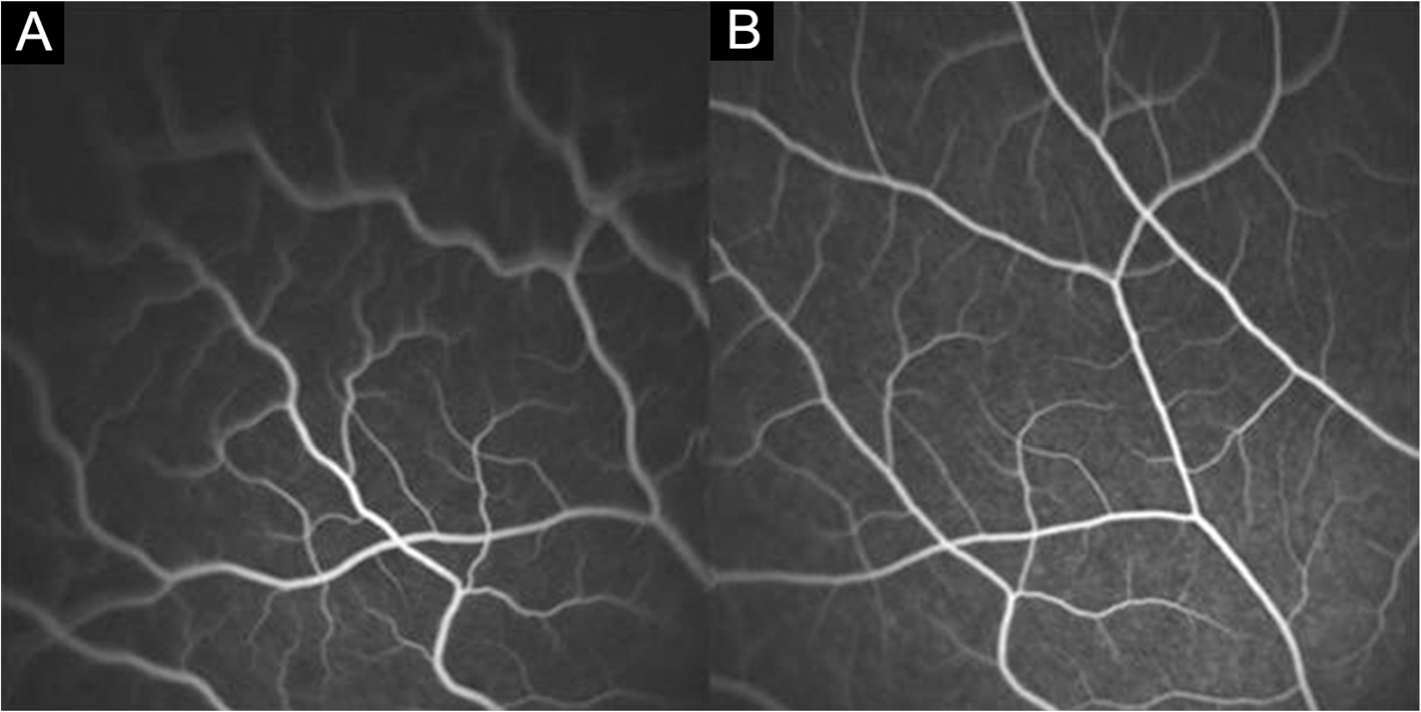
**a** Fluorescein angiography (FA) of case 1 shows increased tortuosity of the mid-peripheral retinal vessels in the acute stage. This vascular tortuosity may result from peripheral choroidal effusion. **b** After 1 month, the vascular tortuosity disappears

***Tortuous and dilated retinal vessels:*** FA and ICGA showed dilated major branches of retinal vessels (Fig. 1a) and tortuosity of mid-peripheral retinal vessels (Fig. 5a). These signs were found in case 1 and 2.

### UBM and ultrasonography findings

**Fig. 6:**
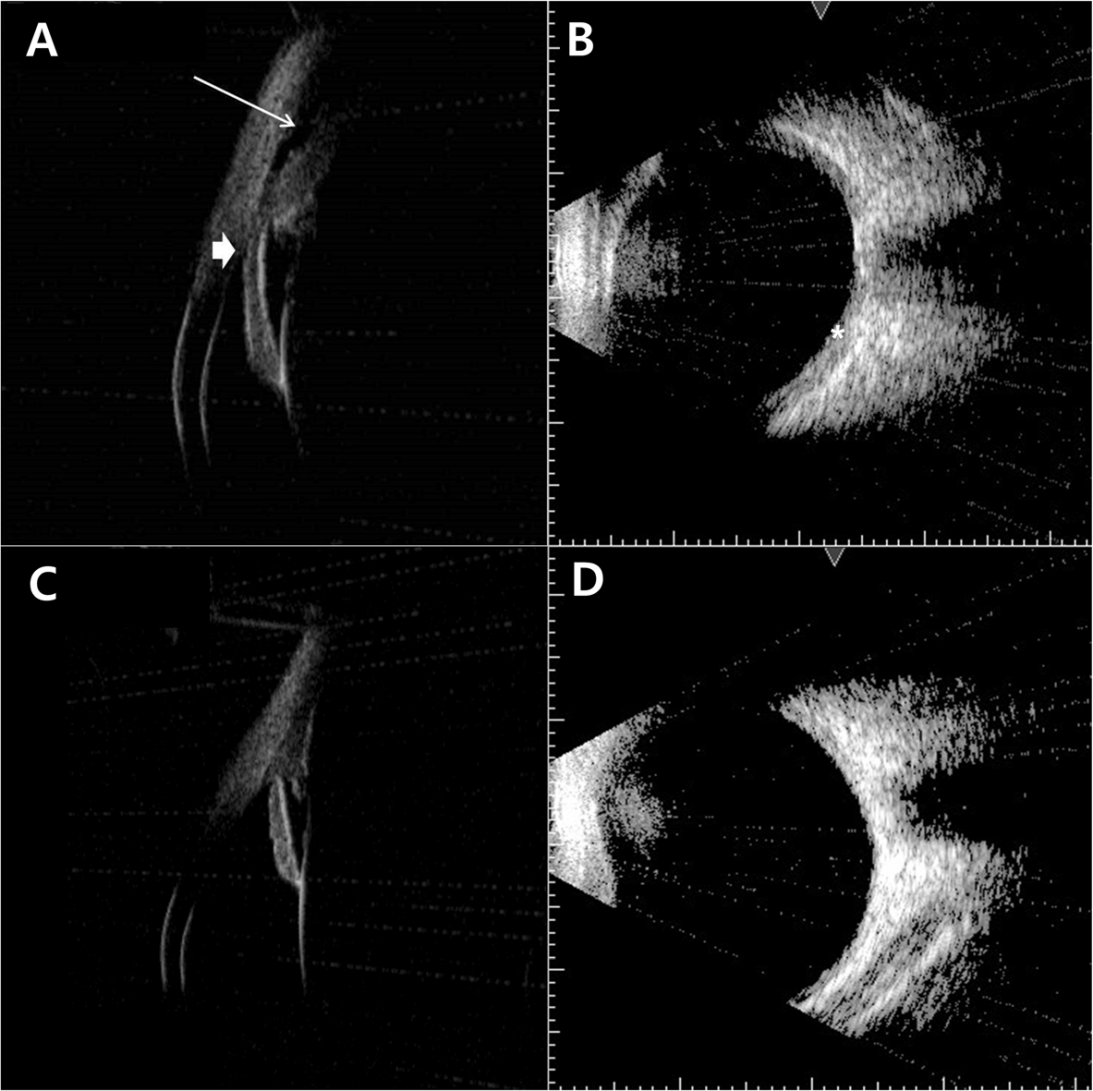
**a** The initial ultrasound biomicroscopy (UBM) shows angle closure with shallow anterior chamber (thick arrow) and anterior rotation of the ciliary process with ciliochoroidal effusion (thin arrow). **b** B-scan ultrasonography shows diffuse thickening of the choroid (asterisk). **c** One month after treatment, UBM of the left eye shows normal anterior chamber angle and disappearance of ciliochoroidal effusion. **d** B-scan ultrasonography shows no choroidal thickening

Initial UBM showed angle closure with shallow anterior chamber and anterior rotation of the ciliary process with ciliochoroidal effusion in case 2 (Fig. 6a). B-scan ultrasonography showed diffuse thickening of the choroid (Fig. 6b). One month after treatment, UBM shows normal anterior chamber angle and disappearance of ciliochoroidal effusion (Fig. 6c). B-scan ultrasonography shows no choroidal thickening (Fig. 6d).

## Discussion

Drug-induced bilateral acute angle closure with ciliochoroidal effusion is both a commonly reported and dangerous side effect [14]. Sulfa drugs have been reported to cause transient myopia, uveal effusion, ciliary body edema, and anterior rotation of the lens-iris diaphragm [6]. Until recently, topiramate, a commonly used antiepileptic drug, has been reported as a cause of secondary angle closure [1–3]. Carbonic anhydrase inhibitors, such as acetazolamide and methazolamide, are systematically administered agents in glaucoma therapy. Paradoxically, there are some reports of angle closure with ciliochoroidal effusion caused by acetazolamide and methazolamide [6–9]. Cases 3, 4 and 5 also have resulted from exposure to these sulfonamide-containing drugs and case 4 was reported as case report in our previous article [8]. Recently, Vishwakarma et al. [11] reported a case of transient myopia and angle closure glaucoma after mefenamic acid medication. The case 1 patient is the second report of angle closure and transient myopia induced by mefenamic acid ingestion. Lee et al. [12] reported a case of acute transient myopia with ciliochoroidal effusion induced by phendimetrazine and ephedrine. The case 2 patient is the second case report of transient myopia and angle closure after ingestion of anorexiants, including phendimetrazine and ephedrine.

In our case series, all patients showed the same clinical manifestations after ingestion of different drugs. All patients showed bilateral angle closure and transient myopia without inflammatory signs such as anterior chamber cells or vitreous cells. Diffuse choroidal thickening was found using B-scan ultrasonography, and ciliochoroidal effusion with ciliary body edema was found in UBM. All patients showed similar angiographic findings in FA and ICGA. In addition, rapid clinical improvement occurred in all patients after discontinuation of suspected drugs. These findings suggest that the pathogenesis of drug-induced angle closure and transient myopia have the same mechanism, although the causative drugs were different.

The exact mechanism of drug-induced bilateral acute angle closure and transient myopia is not fully understood. Krieg et al. [15] reported drug-induced ciliary body edema with transient myopia following exposure to sulphonamide diuretics. They postulated that sulphonamide diuretics stimulate the synthesis of prostaglandin E2 which can cause vasodilation with increased permeability and make ciliary body, retinal, and choroidal edema with breakdown of blood-aqueous barrier.

Ikeda et al. [16] suggested a disease entity called ciliochoroidal effusion syndrome which may occur after unknown postoperative mechanisms, ocular inflammation, systemic diseases, venous congestion, trauma, or drugs such as sulfa derivatives. They suggested ciliochoroidal effusion syndrome result from anterior rotation of the ciliary body due to ciliochoroidal effusion, and subsequent anterior shift of the lens–iris diaphragm. In addition, circulatory disturbances in the choroid and increased permeability of the choroidal vessels may play a role in the development of the ciliochoroidal effusion [17]. Our case reports with ICGA findings are consistent with this hypothesis.

The mechanisms by which ciliochoroidal effusion and ciliary body edema cause acute angle closure and transient myopia have been characterized using UBM [1, 3, 8, 9, 16]. Ciliary body edema and anterior choroidal effusion lead to anterolateral rotation of the ciliary processes around the scleral spur, pushing the lens-iris diaphragm forward and obliterating the ciliary sulcus. These mechanisms result in a shallow anterior chamber and myopic shift. We noticed very similar findings using UBM in all cases of the present study and suggest that ciliary body edema and ciliochoroidal effusion may lead to bilateral angle closure and transient myopia. We found a difference of degrees in myopic shifting in each eye of case 3 and presumed that because the right eye was pseudophakic and the left eye remained phakic, each eye could have differences in the movements of the lens-iris diaphragm.

Blain et al. [10] first reported FA findings in drug-induced annular choroidal detachment and transient myopia in a 38-year-old male who developed − 5 D of myopia after indapamide medication. They reported scattered islands of delayed filling of fluorescence in the early and midphase of FA and postulated that the transient lobular choriocapillaris hypoperfusion was related to choroidal thickening. These hypofluorescent spots were same as HDS seen in our cases. However, in our cases, HDS were not transient and persisted into the late phase of FA and ICGA.

The ICGA findings of our cases share some similarities with those of Vogt-Koyanagi-Harada disease (VKH). Herbort et al. [18] reported ICGA findings of VKH and classified them into four major groups that included (1) early choroidal stromal vessel hyperfluorescence and leakage, (2) hypofluorescence dark dots, (3) fuzzy vascular pattern of large stromal vessels, and (4) disc hyperfluorescence. They also reported three minor signs such as disturbance/delay in early choriocapillaris circulation, hyperfluorescent pinpoints, and exudative subretinal hyperfluorescence and diffuse late hyperfluorescence. In our case series, hypofluorescent dark spots were found in all cases. Hyperfluorescent pinpoints were also seen in our cases, but they did not show any leakage. Early choroidal stromal vessel hyperfluorescence was found in two cases and diffuse choroidal hyperfluorescence in the intermediate phase was found in two cases. Other findings such as fuzzy vascular patterns of large stromal vessels, disc hyperfluorescence, disturbance/delay in early choriocapillaris circulation, and exudative subretinal hyperfluorescence were not seen in our cases. There are several reported cases of acute angle closure as a first manifestation of VKH [19, 20]. The most important difference of our cases from VKH was the absence of exudative retinal detachment and inflammatory signs such as vitreous or anterior chamber cells.

HDS in the ICGA were major angiographic findings and were found in all cases. HDS were small and round in the posterior pole but large and linearly shaped in the peripheral retina (Figure 1b, 2b, c). Different shapes of HDS were due to the differences of choriocapillaris distribution. The choriocapillaris showed dense, honeycomb-like, nonlobular structure in the submacular and peripapillary areas and lobular-like pattern in the posterior pole and equatorial areas. Choriocapillaris formed a more elongated palm-like vascular network in the peripheral areas [21].

Some HDS in our case series became isofluorescent in the mid- to late phase of ICGA, but some HDS remained hypofluorescent throughout the late phage. These differences may be due to the degree of extravascular transudate resulting from disturbed vascular permeability of the choroid. Relatively large amounts of fluid accumulation, which occupied the whole thickness of choroid, caused HDS to be present up to the late phase. Small amount of fluid accumulation, which did not occupy the whole thickness of the choroidal layer, caused the HDS to become isofluorescent in the late phase.

HDS in the ICGA have also been observed in other disorders such as nanophthalmos, uveal effusion syndrome, posterior scleritis, angioid streaks, and a rare case of central serous chorioretinopathy [22–26]. HDS in our case series were different from other disorders in that there were no fundus changes such as Leopard spots and no permanent retinal pigment epithelial changes.

The pinpoint hyperfluorescence in the late phase of ICGA in our cases has also been observed in inflammatory diseases such as VKH or posterior scleritis [18, 23]. In VKH, hyperfluorescent pinpoints are usually found within large areas of hypofluorescence, which often correspond to areas of neurosensory retinal detachment. However, hyperfluorescent pinpoints in our case series were found near HDS and did not show leakage. Therefore, these findings are not evidence of active focal inflammation combined with leakage. However, because the lesions completely resolved in the convalescent stage, they could be a sign of acutely reversible alterations to the choroidal vasculature.

We defined three minor signs of angiography. Hyperfluorescence and leakage from choroidal stromal vessels in the early phase were seen in case 1 and 3 (Figure 3a, b). Diffuse hyperfluorescence of the choroid was seen in the intermediate phase in case 1 and 2 (Figure 4). These findings were very similar to the ICGA findings of VKH. Tortuous and dilated retinal vessels in FA and ICGA (Figure 1a and 5a) could result from peripheral choroidal detachment. Peripheral retinal vascular leakage in FA was found only in case 2 (Figure 2a), but in other cases, retinal vasculature was not altered in FA and ICGA.

Numerous studies have reported that drug-induced bilateral angle closure with ciliochoroidal effusion is a self-limiting and transient condition [1–12, 14]. The most important and the first step in treating this condition is the early recognition and discontinuation of the causative drugs. IOP-lowering medications and high-dose steroids could be used to control the IOP and suppress the reactive metabolites related to the unwanted reactions. Laser or surgical interventions should be avoided, because peripheral iridectomy is ineffective and this condition can be effectively managed in a conservative manner with cycloplegics. In addition, miotics can aggravate the appositional closure of the peripheral angle resulting from the forward movement of the lens-iris diaphragm.

## Conclusions

In conclusion, acute drug-induced angle closure with choroidal effusion is idiosyncratic reaction in the uveal tissue to systemic medications that causes extravascular fluid accumulation in the choroidal layer. This transient condition can be rapidly resolved by prompt recognition and subsequent drug discontinuation. Angiographic findings are consistent with the hypothesis that the main pathophysiological events occur within the ciliochoroidal layer, and that transient extravascular fluid accumulation in the choroid and ciliary body plays a major role in the pathogenesis.

In future studies, angiography and enhanced depth imaging optical coherence tomography could help to characterize the pathophysiological entities of this condition by lesion-specific imaging of the choroidal layer.

## Data Availability

The datasets used and/or analyzed during the current study available from the corresponding author on reasonable request.

## Abbreviations

BCVA: Best corrected visual acuity
D: Diopter
FA: Fluorescein angiography
HDS: Hypofluorescent dark spots
ICGA: Indocyanine green angiography
IOP: Intraocular pressure
UBM: Ultrasound biomicroscopy
VKH: Vogt-Koyanagi-Harada disease
